# Capture at the single cell level of metabolic modules distinguishing aggressive and indolent glioblastoma cells

**DOI:** 10.1186/s40478-019-0819-y

**Published:** 2019-10-16

**Authors:** Mirca S. Saurty-Seerunghen, Léa Bellenger, Elias A. El-Habr, Virgile Delaunay, Delphine Garnier, Hervé Chneiweiss, Christophe Antoniewski, Ghislaine Morvan-Dubois, Marie-Pierre Junier

**Affiliations:** 1 CNRS UMR8246, Inserm U1130, Sorbonne Université, Neuroscience Paris Seine-IBPS, Team glial plasticity and neurooncology, Paris, France; 2 ARTbio Bioinformatics Analysis Facility, Sorbonne Université, CNRS, Institut de Biologie Paris Seine, 75005 Paris, France; 3 Cell Death and Drug Resistance in Lymphoproliferative Disorders Team, Centre de Recherche des Cordeliers, Sorbonne Université, INSERM UMRS 1138, 75005 Paris, France

**Keywords:** Glioma, Computational modeling, scRNA-seq, PUFA, Elongase

## Abstract

Glioblastoma cell ability to adapt their functioning to microenvironment changes is a source of the extensive intra-tumor heterogeneity characteristic of this devastating malignant brain tumor. A systemic view of the metabolic pathways underlying glioblastoma cell functioning states is lacking. We analyzed public single cell RNA-sequencing data from glioblastoma surgical resections, which offer the closest available view of tumor cell heterogeneity as encountered at the time of patients’ diagnosis. Unsupervised analyses revealed that information dispersed throughout the cell transcript repertoires encoded the identity of each tumor and masked information related to cell functioning states. Data reduction based on an experimentally-defined signature of transcription factors overcame this hurdle. It allowed cell grouping according to their tumorigenic potential, regardless of their tumor of origin. The approach relevance was validated using independent datasets of glioblastoma cell and tissue transcriptomes, patient-derived cell lines and orthotopic xenografts. Overexpression of genes coding for amino acid and lipid metabolism enzymes involved in anti-oxidative, energetic and cell membrane processes characterized cells with high tumorigenic potential. Modeling of their expression network highlighted the very long chain polyunsaturated fatty acid synthesis pathway at the core of the network. Expression of its most downstream enzymatic component, ELOVL2, was associated with worsened patient survival, and required for cell tumorigenic properties in vivo. Our results demonstrate the power of signature-driven analyses of single cell transcriptomes to obtain an integrated view of metabolic pathways at play within the heterogeneous cell landscape of patient tumors.

## Introduction

Glioblastoma (GBM), the most common form of malignant brain tumors in adults, are characterized by extensive cell heterogeneity. This cell heterogeneity results from irreversible processes such as clonal selection of distinct mutations and differentiation of cancer stem cells, but also from the cells’ ability to adapt their functioning to variations in their environment and to therapies [2, 13, 34]. As a result, cancer cells coexist within GBM micro-territories in various functioning states, with respect to stem-like, proliferation, migration, pro-angiogenic, drug resistance, or tumor-initiating (i.e. tumorigenic) capacities [9, 16, 49, 70]. Such heterogeneity in cell functioning defies therapeutic targeting.

**Fig. 1:**
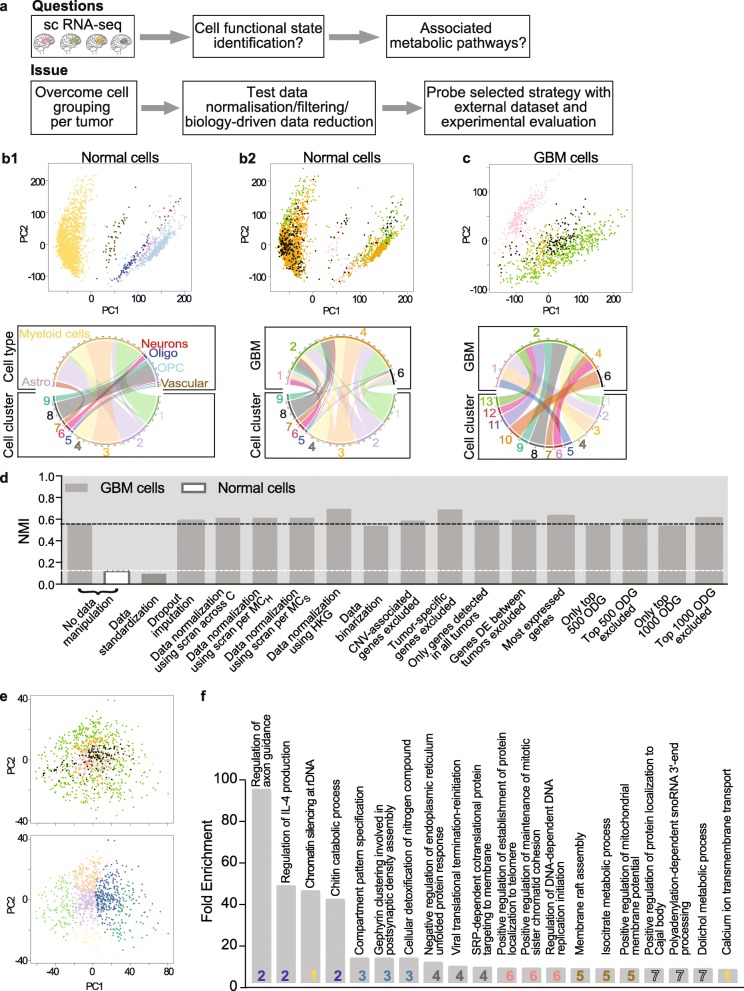
Spontaneous grouping of cancer cells by tumor of origin following unsupervised analysis. **a** Analytical and experimental strategy outline. **b** Normal cells group independently from tumor of origin. PCA (top) and chord (bottom) plots. Each dot represents a cell in PCA. b_1_: cells colored by normal cell type identity (purple: astrocytes; blue: oligodendrocytes; light blue: oligodendrocyte precursor cells; red: neurons; gold: myeloid cells; brown: vascular cells). Normal cell types determined as described [14]. b_2_: cells colored by tumor of origin (pink, green, orange, black for GBM1, 2, 4 and 6, respectively). **c** Cancer cells group by their tumor of origin. PCA (top) and chord (bottom) plots. Cells colored by tumor of origin (pink, green, orange, black for GBM1, 2, 4 and 6, respectively). **d** Impact of data treatment on the dependence of cell clustering to tumors. NMI: Normalized Mutual Information score. C: cells. MC_H_: metacell defined by hierarchical clustering. MC_S_: metacell defined by SNN (shared nearest neighbor) clustering. HKG: housekeeping genes. CNV: copy number variations. DE: differentially expressed. ODG: overdispersed genes. Black and white dotted lines: reference NMI scores of grouping analyses performed with all genes detected in GBM and normal cells, respectively. Note that NMI scores of GBM cell grouping remain constant, regardless of data normalization or filtering modes. Only data standardization reduces NMI score to a value similar to that obtained when analyzing normal cells. **e** Unsupervised analysis of data standardized by tumor results in clusters mixing cells from different tumors. PCA plots highlighting the tumor from which the cells derive (top: pink, green, orange, black for GBM1, 2, 4 and 6, respectively) or the 7 clusters identified (bottom) . **f** Gene ontology analysis of the genes describing each of the 7 clusters highlights a variety of biological processes, not linkable to specific functioning states. DAVID toolkit. Corresponding cluster number is indicated (colored as cluster colors in the bottom panel of e)

The changes in cell functioning state are accompanied by variations in cell metabolic activities. These variations are essential for GBM cells to exploit different sources of nutrients such as glucose, glutamine or acetate, and thereby cope with changes in oxygen and nutrient availabilities that occur throughout tumor development [47, 48, 52]. The significance of these metabolic variations for the cell behavior may extend beyond a passive response to environmental signals, as recent evidence support a role for metabolism as a driver of changes in cell functional status. Flavahan and colleagues demonstrated that upregulation of the high-affinity glucose transporter GLUT3 promotes acquisition by GBM cells of tumorigenic properties [24]. Conversely, we found that decreased activity of the mitochondrial enzyme SSADH triggers GBM cell conversion into a less aggressive functioning state, by coupling enhanced levels of the GABA by-product GHB to altered epigenetic regulations [19]. These metabolic variations have been found to take place within the patient tumors, and to be coherently linked with relevant phenotypic markers [19], or patients’ clinical course [12, 24]. Metabolism is also emerging as a player in GBM therapeutic resistance, as exemplified by escape from the anti-angiogenic Bevacizumab treatment. This escape has been linked to an increase in glycolysis and its uncoupling from oxidative phosphorylation in favor of lactate production in in vivo GBM models as well as in patients [20]. Metabolic enzymes are at the core of the molecular pathways controlling cell functioning states. Correcting their deregulation is therefore expected to be efficient to prevent acquisition and maintenance of aggressive cell functioning states shared by cell subpopulations in all GBMs, regardless of their genomic specificities. Exploitation of metabolic targeting for therapies demands therefore to identify the metabolic pathways at play within the patient tumors in link with the heterogeneity of cell functioning states observed in GBMs. Here, we used publicly available GBM single cell RNA-sequencing (scRNA-seq) data from four patients with *EGFR* amplification [14] for identifying metabolic pathways prevailing in GBM cell subpopulations in their most aggressive functioning state (Fig. 1a).

Transcriptomes obtained by scRNA-seq are endowed with the potential to deliver information on a cell functioning state and its underlying molecular networks. Analyses of scRNA-seq from different tumors using current methods result in the predominant grouping of cancer cells according to the tumor from which the cells are isolated (hereafter designated as tumor of origin). In contrast, normal cells present in the tumor are grouped according to their lineage subtype (e.g. neural, immune, vascular), regardless of their tumor of origin [14, 57, 64]. Characterizing the source of this specific influence of the tumor of origin on cancer cell grouping led to the development of a data reduction approach based on a molecular signature for identifying cell functioning states. Combining analyses of GBM unicellular and tissue transcriptomes with experimental assays, we highlighted a combination of metabolic pathways prevailing in cells with high tumorigenic potential. The analytical method developed is provided.

## Material and methods

All figures were prepared using Adobe Illustrator (Adobe Systems). All bioinformatics analyses were performed using the R software version 3.5.0 (https://cran.r-project.org/). Detailed methods are provided in Additional file 1. All resources and materials, R packages, corresponding websites and references are listed in Additional file 2.

### Computational analyses

Single cell transcriptomes of 1033 GBM and 2417 normal cells from four patients were used [14]. This dataset distinguishes cancer from normal cells on the basis of chromosome copy number variations (CNV) profiling, and further distinguishes normal cells according to their neural or immune lineage subtype [14]. We filtered out low complexity transcriptomes on the basis of the graphical distribution of the number of transcripts and genes per cell (Additional file 3: Figure S1A and S1B). Cells with fewer than 90,000 transcript reads and fewer than 1700 detected genes were filtered out (Additional file 3: Figure S1A and S1B). We used log2-transformed Counts Per Million (log2(CPM + 1)) to allow comparison of read abundance across libraries of different sizes, unless otherwise specified. In an analysis subset, tumor-per-tumor data standardization was achieved by centering and reducing the data on a gene-by-gene basis (Additional file 4, [8, 45]). Normalization on the basis of the expression of a set of 17 housekeeping genes (HKG) is detailed in Additional file 1. Tissue transcriptomes corresponded to the TCGA RNA-seq dataset of 155 untreated GBM patients [5] (normalized counts, log2(TPM + 0.5), Additional file 2). For comparison purposes, we also used single cell transcriptomes of the 4916 GBM cells derived from 20 GBMs operated from adult patients, which became available during the reviewing process of this article [51]. Gene expression data currently available for this dataset are in log2((TPM/10) + 1). TPM (Transcripts Per Million) normalization takes into account an eventual biased estimation of long transcript numbers, by dividing the number of mapped reads by the transcript’s length. Since we could not calculate expression values in CPM + 1 from these data, we just transformed the log2((TPM/10) + 1) into log2(TPM + 1) to optimize the comparison between the Darmanis [14] and Neftel datasets [51]. Grouping analyses were performed using the Hierarchical Clustering on Principal Components (HCPC) approach (Additional file 4). Results were visualized using Principal Component Analysis (PCA) or t-distributed Stochastic Neighbor Embedding (tSNE). HCPC was also used to identify genes whose mean expression in one cluster differs from their mean across all cells (i.e. variables driving cell grouping). Normalized Mutual Information (NMI) scores were calculated to determine the contribution of cells issued from distinct tumors to each cluster. A NMI value of 1 implies that clusters gather objects (here, cells) corresponding to a single label (here, the tumor label) whereas a value of 0 denotes that all labels are split across all clusters (Additional file 2, [46]). R scripts used for unsupervised grouping and associated analyses are provided in Additional file 4. Tumorigenic and other scores were obtained by computing the geometric mean of the expression values per cell of each element of the molecular signature corresponding to a given score. When null, expression values were imputed a value of 1. Each element of the signature was detected in at least 25% of GBM cells. Gene differentially expressed between cell or tissue groups with differing scores were identified following Mann-Whitney (Wilcoxon Rank Sum) test with *p*-values adjusted for multiple testing (Benjamini-Hochberg (BH), *p*-value < 0.01) [62]. Gene ontology analysis was done using the human genome as background (Additional file 2). Functional gene network reconstruction was achieved using the information-theoretic method, MIIC (multivariate information-based inductive causation, Additional file 1 [61, 69]). Four independent datasets comprising 153 to 485 primary GBM transcriptomes were used for patient survival analysis (Additional file 2). Construction of a principal curve was achieved with a PCA based on the expression of each component of the lipid subgroup (16 genes coding for enzymes of the lipid metabolism overexpressed in Tum^HIGH^ cells and tissues and detected in at least 25% of GBM cells, see results). Each cell was projected onto the curve using the Pathifier algorithm (Additional file 2, [18]). Lipid subgroup and vesicle scores (Additional file 2) were calculated as described for the tumorigenic score.

### Biological experiments

Patient-derived cells (PDC) 6240**, R633 and 5706** were obtained from neurosurgical biopsy samples of distinct primary GBMs, characterized and cultured as described [19, 60]. Lentiviral transduction (Additional file 2) was achieved as described [4, 19]. Viable cell counting, gene expression analysis, intracranial xenografts, and bioluminescence imaging (Additional file 2) were performed as described [4, 19]. All experiments were performed using independent biological samples, each independently repeated at least three times with the exception of the xenograft experiments. Prism 7.0 software (GraphPad) was used for statistical analyses.

### Statistical analyses

Statistical analyses were performed with significance level set at *p* < 0.05, except for differential gene expression analysis for which a *p* < 0.01 was chosen to reduce the chances of false positives. The type of statistical test and *p*-values are provided in the figure legends.

## Results

### Unsupervised clustering analysis highlights first GBM cells’ tumor of origin

We used the publicly available single cell transcriptome dataset from Darmanis and colleagues [14] after removing low-complexity cell transcriptomes. Genes detected in at least 3 transcriptomes were retained for analysis (18,577 genes for GBM cells and 19,699 genes for normal cells). Previous scRNA-seq analyses of GBMs and other cerebral tumors focused on the most dispersed [14] or most expressed genes [23, 53, 65, 68] or on meta-modules of genes revealed by hierarchical clustering of cells tumor per tumor and recurring in several tumors [51]. These analyses resulted in the identification of cell lineages and cell genomic anomalies rather than cell functioning states. We chose therefore to conserve all potential information by analyzing the full set of selected genes.

Gene expressions were computed as log2(CPM + 1). Hierarchical Clustering on Principal Components (HCPC) of gene expressions in the mixed pool of GBM and normal cells readily separated cancer from normal cells (Additional file 3: Figure S1C). This result obtained by analyzing all detected genes is similar to the one obtained previously by analyzing the top 500 overdispersed genes [14]. Separate HCPC of normal cells resulted in six cell groups of immune or neural subtypes (astrocytes, oligodendrocytes, oligodendrocyte precursor cells, neurons, myeloid cells, vascular cells, Fig. 1b1), each group mixing cells from different tumors (Fig. 1b2). In striking contrast, GBM cell clusters resulting from HCPC analysis were dominated by cells from a single patient tumor (Fig. 1c). Contribution of the different tumors to clusters was scored by computing Normalized Mutual Information (NMI) between clusters and tumor labels, NMI scores being expected null if each tumor contributes equally to each cluster (Additional file 1). Fully supporting our observation, the NMI score of normal cell clustering was only of 0.12 whereas the one of tumor cells was of 0.55 (two first bars in Fig. 1d). This predominant grouping of cancer cells by their tumor of origin has been reported for a number of cerebral and non-cerebral tumors [11, 14, 23, 35, 39, 57, 64]. For identifying traits common to all tumors, data can be analyzed tumor per tumor [65, 67, 73], or merged and analyzed as a whole after standardization (i.e. subtracting from each expression value the gene expression mean and dividing by its standard deviation across cells within a given tumor) [50]. The numbers of cancer cells precluding confident per-tumor analysis, we turned to standardization. HCPC analysis of standardized data resulted in clusters mixing cells from different GBMs (Fig. 1e), as shown by a NMI score similar to the one calculated for normal cell grouping (third bar in Fig. 1d). However, gene ontology analysis of the genes identified in the HCPC as driving the cell grouping (see methods) did not provide clear links between cell clusters and potential cell functioning states (Fig. 1f, Additional file 5).

These results prompted us to further explore non-standardized data for minimizing the factors that might account for predominant grouping of cancer cells according to their tumor of origin.

### Tumor identity encoded by information dispersed through GBM cell transcript repertoires

Two main factors can account for tumor-driven cell grouping: the technical variations in tumor sample scRNA-seq, collectively referred to as batch effect, and the biological tumor-specific variations.

**Fig. 2:**
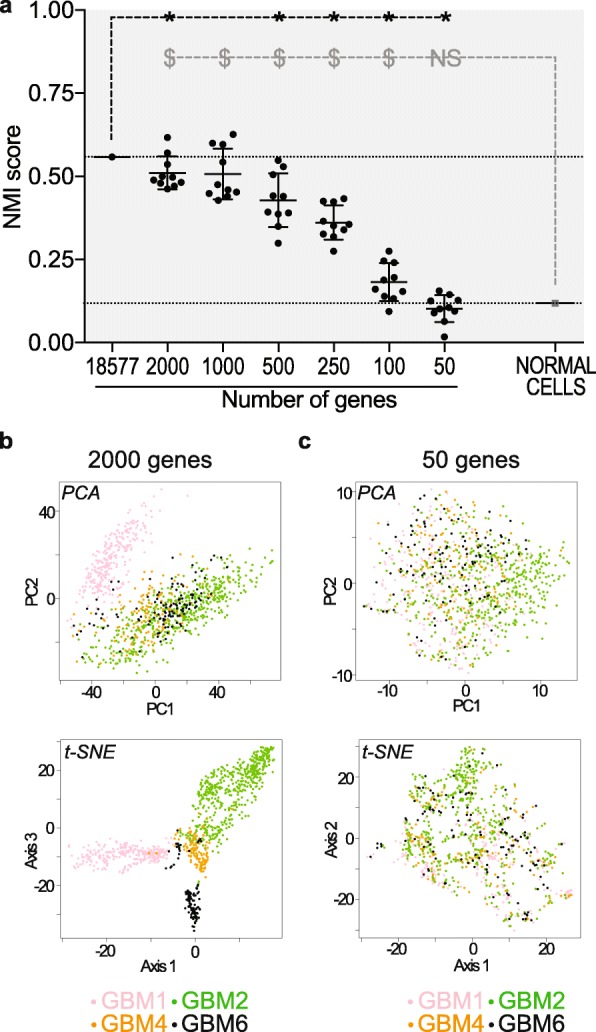
Down-sampling gene numbers relieves tumor-driven cell grouping. **a** Decreased Normalized Mutual Information (NMI) score when reducing gene numbers used for grouping analyses. Ten independent analyses performed with randomly selected genes for each gene number analyzed. Mean ± SD. One-sample t-test. * *p* < 0.01 compared to the NMI score of the grouping analysis performed with all genes detected in GBM cells. $ *p* < 0.0001 compared to the NMI score of the grouping analysis performed with all 19,699 genes detected in the dataset of normal cells. Note that NMI scores consistently decrease below 500 genes analyzed, reaching values similar to the NMI score of the grouping analysis of normal cells only in grouping analyses performed with 50 genes. **b** Example of a cell grouping analysis using 2000 randomly sampled genes. The clusters are predominantly composed of cells from a single tumor. Each dot represents a cell colored according to its tumor of origin. PCA and tSNE visualizations in upper and lower panels, respectively. **c** Example of a cell grouping analysis using 50 randomly sampled genes. Cells from a given tumor are distributed in different clusters. Each dot represents a cell colored according to its tumor of origin. PCA and tSNE visualizations in upper and lower panels, respectively

In scRNA-seq experiments, batch variations in RNA quality and sequencing efficiency, regardless of their origin, translate into variations in sample-dependent gene detection failures (referred to as dropouts) and in sample sequencing depth. Grouping of normal cells independently from their tumor of origin indicates that such batch variations are minor. We tested the influence of dropouts [44] and of an additional normalization of the sequencing depth using a set of GBM-specific housekeeping genes (HKG) on the cell grouping (Additional file 6). We also tested normalization based on the scran method, which calculates scaling factors for small homogeneous groups of cell libraries [66]. Neither dropout imputation, nor scran normalization, nor HKG normalization corrected tumor-driven cell grouping (Fig. 1d, Additional file 3: Figure S2A-E). These results confirmed that batch effects are not major contributors of this grouping. Inter-tumor biological differences encompass genomic alterations known to vary greatly from one GBM to another, the tumor developmental stage, or the brain area and/or cells from which it developed [58]. We reasoned that differing biological characteristics, whatever their source, would translate into gene repertoires differing between tumors. To test this possibility, we considered binarization of the data by applying a value of one to all expressed genes regardless of their relative expression levels, and zero for non-detected ones. Maintenance of cell grouping by tumor of origin following binarization of gene expression (Fig. 1d and Additional file 3: Figure S2F) showed that cell gene repertoires are more similar within a given tumor than between two different tumors. We therefore sought to better understand which genes contribute most to this variability between cell transcriptomic landscapes. We first tested the impact of chromosome CNV on the cell grouping by filtering out genes mapped to chromosomes with CNV as previously identified [14]. Taking into account CNV did not modify tumor-driven cell grouping (Fig. 1d and Additional file 3: Figure S2G). Likewise, excluding genes detected in a single tumor or including only genes detected in all tumors did not change the outcome of HCPC analyses (Fig. 1d and Additional file 3: Fig. S2H and I). We then tested the influence of inter-tumor variability in gene expression. Exclusion of the 100 genes identified as differentially expressed between tumors by Darmanis and colleagues [14] did not modify the outcome of the analysis (Fig. 1d and Additional file 3: Figure S2J). We then considered the most expressed genes, calculating the aggregate expression of each gene across cells, and retaining genes with the highest aggregate expression as described [65]. HCPC using the resulting 9505 most expressed genes still grouped cells by their tumor of origin (Fig. 1d and Additional file 3: Figure S2K). Likewise, performing HCPC using the top 500 or 1000 genes with expression variability between cells higher than expected (i.e. overdispersed [21]), or after excluding them, resulted in a similar tumor-driven cell grouping (Fig. 1d and Additional file 3: Figure S2L-O). Altogether, these results indicate that tumor-driven cell grouping is not based on limited and tumor-specific sets of genes. This led us to envisage that primary grouping of cancer cells by tumor of origin could result from information dispersed throughout the whole cell transcriptome. We challenged this hypothesis by performing iterative HCPC analyses on decreasing numbers of genes randomly selected among the 18,577 detected genes. Ten analyses of distinct sets of randomly selected genes were performed for each size of gene sets (*n* = 2000, 1000, 500, 250, 100, and 50). NMI scores of the clusterings remained unchanged for gene set sizes > 500 (Fig. 2a and b). Their gradual decrease below this threshold indicated a progressive reduction of the influence of the tumor of origin on cell grouping. This influence was suppressed only when reducing the number of analyzed genes to 50, as shown by NMI scores equivalent to that calculated from the grouping analysis of the 19,699 genes detected in normal cells (Fig. 2a and c). Altogether, these results show that tumor-driven cell grouping is irreducible to differential expression of circumscribed gene groups. To the contrary, it is encoded by information dispersed throughout the cell transcript repertoires, which is retrieved as soon as a combination of expressions of more than 500 genes is included in the analyses. As a consequence, unsupervised analysis turns out to be inadequate for identifying cell functioning states common to all tumors. We thus turned towards an approach of data reduction based on a signature of a functionally coherent set of genes.

### GBM cell grouping according to their tumorigenic potential upon signature-driven reduction of scRNA-seq data

We developed a grouping method based on a molecular signature we previously identified [4]. The signature is composed of five transcription factors, ARNT2, POU3F2, OLIG2, SOX9 and SALL2, with co-varied expression in GBM tissues and cells [4]. ARNT2 binding sites were identified by chromatin-immunoprecipitation assays in regulatory elements of the *POU3F2*, *OLIG2* and *SOX9* genes that were found to be downregulated upon *ARNT2* knockdown [4]. Each of the five signature elements was demonstrated to be required for GBM cell tumorigenic properties [4, 30, 43, 63]. Altogether, these findings support the participation of these transcription factors to a common regulatory network. We sought to use this signature to highlight subpopulations of cells in a tumorigenic state, expected to be present in all tumors.

**Fig. 3:**
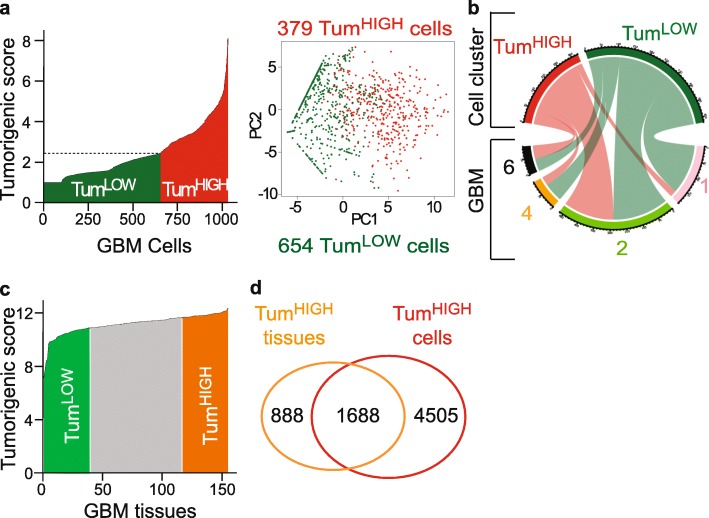
Signature-driven data reduction approach identifies cells according to their potential tumorigenic state. **a** Splitting cells into groups with high (Tum^HIGH^) or low (Tum^LOW^) tumorigenic potential. Left panel: tumorigenic score distribution across the cells. Dotted line: mean of the tumorigenic score. Right panel: PCA plot based on the expression of the 5 elements of the tumorigenic signature. **b** Contribution of each tumor to the two tumorigenic groups identified (chord plot). Note that each tumor contributes to each cell group. **c** Tumorigenic score distribution across GBM tissues (155 GBM tissues, TCGA RNA-seq dataset). Tum^HIGH^ and Tum^LOW^ GBM tissue groups selected at the extreme quartiles of the distribution. **d** High overlap between genes overexpressed in Tum^HIGH^ GBM tissues and cells. 65.5% (1688) of genes overexpressed in Tum^HIGH^ GBM tissues are also overexpressed in Tum^HIGH^ GBM cells

To obtain an index of the cells’ tumorigenicity, we calculated a tumorigenic score corresponding to the geometric mean of the expression of each signature element. The score distribution curve exhibited a main inflection point corresponding to the distribution’s mean (Fig. 3a), which delineated two groups of 654 and 379 cells with low and high tumorigenic scores, respectively, hereafter designed as Tum^LOW^ and Tum^HIGH^ (Fig. 3a). Each of the four tumors contributed to each group (Fig. 3b, NMI score = 0.057). Tum^HIGH^ GBM cells exhibited higher numbers of transcripts and genes than Tum^LOW^ GBM cells (Additional file 3: Figure S3A and B). We identified 6630 genes differentially expressed between both groups (Mann Whitney, BH-adjusted *p*-value < 0.01, Additional file 7), 98% of these genes being overexpressed in Tum^HIGH^ GBM cells. Highly similar results were obtained using data normalized with the scran method (Additional file 7). Of note, several items of the list of genes with enhanced expression in Tum^HIGH^ cells encoded proteins previously implicated in GBM cell aggressiveness (e.g. *E2F1* [71], *EGFR* [1], *NOTCH1* [7], *FABP7* [15], *PTPRZ1* [25]). Conversely, genes with known tumor-suppressor properties were identified among the genes overexpressed in Tum^LOW^ cells (e.g. *TUSC3* [36], *SERPINB1* [33]). The whole workflow is summarized in Additional file 3: Figure S4 and provided in Additional file 8. These results suggest that cell functioning state can be inferred from scRNA-seq data following signature-driven data reduction. We next challenged the relevance of this approach by applying it to an independent dataset and using in vitro and in vivo GBM models.

### Specific amino acid and lipid metabolic pathways distinguish GBM cells and tissues with high tumorigenic potential

**Fig. 4:**
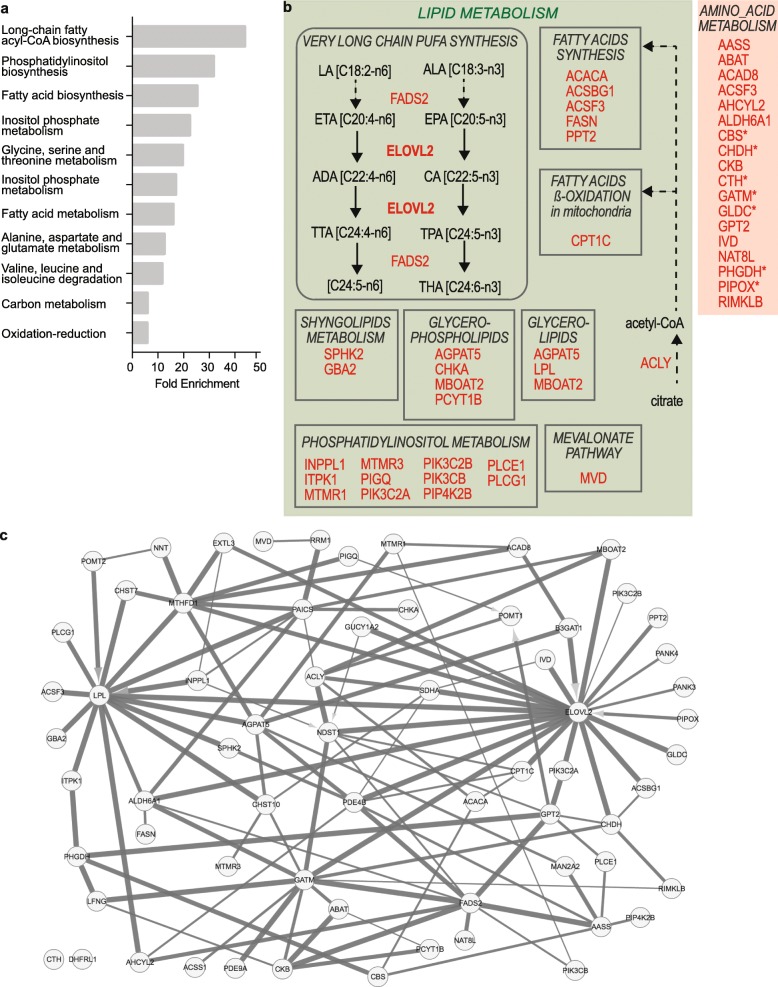
Enrichment in lipid and amino acid metabolism enzymes in Tum^HIGH^ GBM cells and tissues. **a** Gene ontology analysis of the 66 metabolism genes identified among genes overexpressed in both GBM Tum^HIGH^ cells and tissues. DAVID toolkit. **b** Schematic representation of the lipid and amino acid metabolic pathways containing genes overexpressed in Tum^HIGH^ GBM cells and tissues. Asterisks mark genes coding for components of the glycine, serine and threonine metabolism. LA: linoleic acid; ALA: linolenic acid; ETA: eicosatetraenoic acid; EPA; eicosapentaenoic acid; ADA: docosatetraenoic acid; CA: clupanodonic acid; TTA: tetracosatetraenoic acid; TPA: tetracosapentaenoic acid; THA: tetracosahexaenoic acid. **c** Modeling interconnections between the 66 metabolism genes highlights ELOVL2 at the most densely connected node of the network. Gene network built on the basis of the gene expression values across all GBM cells using MIIC tool. Line thickness represents the strength of the edge. Arrowheads linking variables in a v-structure of the type x → y ← z denotes the absence of a graphical structure of the type: x → y → z, x ← y ← z and x ← y → z (variable x cannot be reached passing through y, nor y passing through x, nor y is a common parent of the two other variables). These 3 models can be excluded since in a v-structure y does not mediate mutual information between x and z

To probe the biological relevance of our approach, we first applied the same analytical strategy to an independent dataset. Additional GBM single cell transcriptome datasets equivalent to the one published by Darmanis and colleagues and including several patients were not publicly available prior to submitting this paper for publication. We therefore turned to the TCGA collection of transcriptomes obtained by sequencing the RNA extracted from 155 patients’ GBM tissue fragments. As expected with respect to the heterogeneous nature of GBM tumors where cancer cells with differing properties co-exist with normal neural, vascular and immune cells, we observed a smoother distribution curve of the tumorigenic score across GBM tissues than across GBM cells (Fig. 3c). We therefore used quartiles to delineate two GBM tissue groups with low and high tumorigenic scores, respectively (Fig. 3c), postulating that Tum^HIGH^ tissues contain more Tum^HIGH^ than Tum^LOW^ cells or a cell population expressing very high level of the signature elements. Differential expression analysis between these two groups yielded a list of 6565 genes, 44% of them being overexpressed in Tum^HIGH^ GBM tissues (Mann Whitney, BH-adjusted *p*-value < 0.01, Additional file 7). The list of genes overexpressed in Tum^HIGH^ GBM tissues showed a 65.5% overlap with the list of genes overexpressed in Tum^HIGH^ cells (Fig. 3d, Additional file 7). This result was remarkable considering that it was obtained by confronting a dataset derived from 4 tumors to another derived from 155 tumors, and that tissue transcriptomes correspond to gene expression levels averaged over several hundred thousands of cells. Of the 1688 genes overexpressed in Tum^HIGH^ GBM cells and tissues, 78 encoded metabolic enzymes (Additional file 9, [37]). We further selected the 66 of them significantly correlated to the tumorigenic score across all GBM cells as well as across all GBM tissues (Pearson correlation, p-value < 0.01, Additional file 9). Gene ontology analysis highlighted a 15 to 45-fold enrichment first in components of the lipid metabolism (27 genes) and second in components of the amino acids metabolic pathways (18 genes) (Fig. 4a, Additional file 9 and Additional file 3: Figure S5). Seven of the components of the amino acid metabolism belonged to the glycine, serine and threonine metabolism (Fig. 4b) whereas the lipid metabolism components were distributed among eight subpathways (Fig. 4b). Modeling of the regulatory gene network from the single cell expression data of the 66 metabolism genes using the MIIC algorithm singled out very long chain polyunsaturated fatty acid (VLC-PUFA) synthesis by highlighting ELOVL2 as the densest node of the network (Fig. 4c).

During the reviewing process of this article, a novel and larger dataset of GBM scRNA-seq based also on the SMARTseq2 technology, and with sequencing depth comparable to the one obtained by Darmanis and colleagues became available [51]. We therefore repeated our analysis using the 4916 GBM cell transcriptomes, 2184 coming from GBMs bearing an amplified form of *EGFR* (*EGFR*^AMP^) and 2732 from GBMs bearing a non-amplified *EGFR* (*EGFR*^NON-AMP^). The distribution profile of the tumorigenic score across this set of GBM cells was similar to the one across the Darmanis GBM cell set (Additional file 3: Figure S6A). Cells derived from *EGFR*^AMP^ and *EGFR*^NON-AMP^ GBMs contributed both to the Tum^HIGH^ and Tum^LOW^ GBM cell groups (Additional file 3: Figure S6B). From the list of 6869 genes overexpressed in Tum^HIGH^ cells compared to Tum^LOW^ cells, 60.9% (4185) were also identified as overexpressed in Tum^HIGH^ cells using the Darmanis scRNA-seq data (Additional file 3: Figure S6C). These 4185 genes included 376 genes coding for proteins involved in metabolic pathways and whose expression correlates to the tumorigenic score. Crossing this list of 376 genes with the corresponding list of genes from GBM tissues of the TCGA yielded 59 common genes (Additional file 3: Figure S6D, and Additional file 10). Gene regulatory network reconstruction using MIIC tool highlighted again ELOVL2 at the densest nodes of the network (Additional file 3: Figure S6E-F). These results further strengthen the relevance of our approach. They also show that similar metabolic modules distinguish Tum^HIGH^ and Tum^LOW^ GBM cells independently from *EGFR* amplification.

### Functional association of the lipid metabolism enzyme ELOVL2 to patient clinical outcome and GBM development

**Fig. 5:**
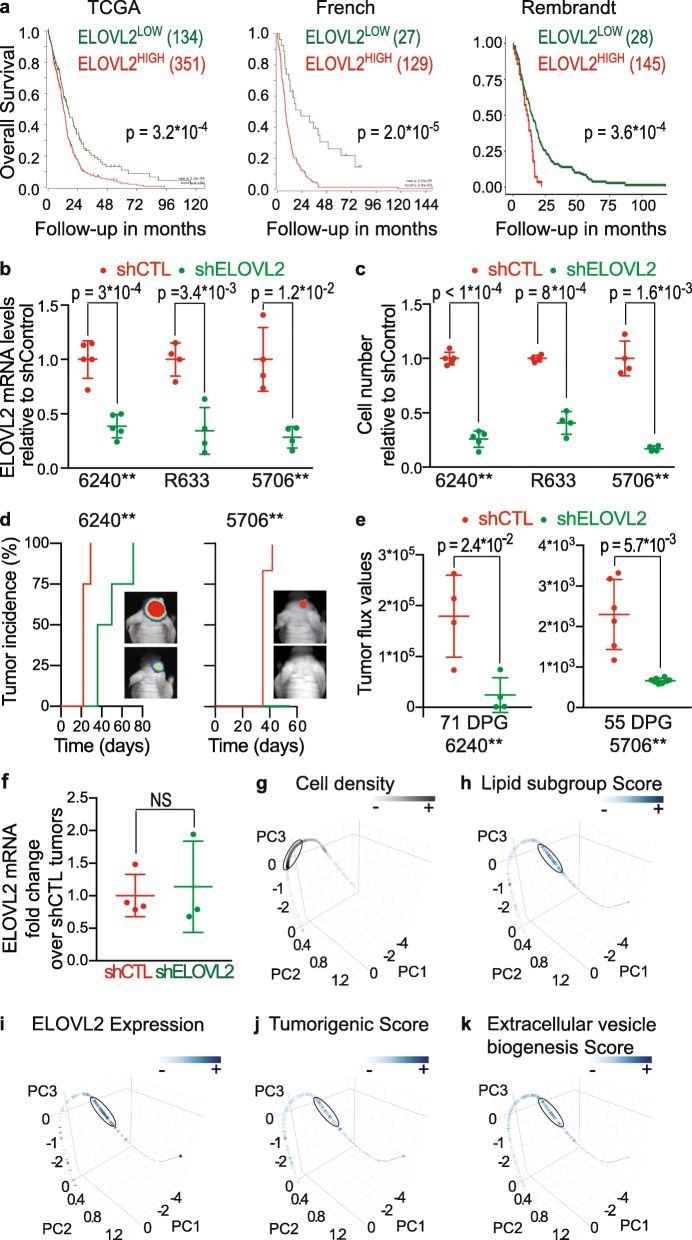
Association of the lipid metabolism enzyme ELOVL2 to patient clinical outcome and GBM development. **a** High *ELOVL2* expression is associated with worse patient survival. GBM tissue transcriptomes (microarrays) of 485, 156 and 173 GBMs of TCGA, French and Rembrandt datasets, respectively. Log-rank (Mantel-Cox) test. **b** Decreased *ELOVL2* mRNA levels in sh*ELOVL2* patient-derived cells (PDC) compared to shControl-PDC. 6240**, R633 and 5706** PDC. QPCR assay. Unpaired t-test with Welch’s correction, mean ± SD, *n* = 4–5 independent biological samples. **c** *ELOVL2* knockdown decreases cell proliferation. 6240**, R633 and 5706** PDC. Unpaired t-test with Welch’s correction, mean ± SD, *n* = 4–5 independent biological samples. **d** *ELOVL2* knockdown delays tumor development. Bioluminescent analyses of tumor growth initiated by grafting PDC transduced with a luciferase construct and either shControl (shCTL) or sh*ELOVL2* constructs. *n* = 4 (6240**) and *n* = 6–8 (5706**) mice per group. **e** *ELOVL2* knockdown decreases tumor burden as shown by quantification of the tumor bioluminescent signals. DPG: days post-graft. *n* = 4 (6240**) and *n* = 6–8 (5706**) mice per group. Mean ± SD. Unpaired t-test with Welch’s correction. Background signal (mean ± SD): 6240** 537 ± 68, *n* = 8, 5706** 589 ± 59, *n* = 14 mice. **f** Recovery of *ELOVL2* expression in tumors forming from xenografts of 6240** sh*ELOVL2*. QPCR assay. Mean ± SD. *n* = 4 for shCTL and *n* = 3 for sh*ELOVL2*. Unpaired t-test with Welch’s correction. **g-k** Principal curve resulting from PCA of the expression of the subgroup of genes encoding lipid metabolism enzymes overexpressed in Tum^HIGH^ cells and tissues. **g** Cell density along the principal curve. The ellipse delineates the portion of the curve with the highest cell density. **h-k** Cells colored according to their (h) score calculated with the components of the lipid subgroup, (i) *ELOVL2* expression levels, (j) tumorigenic score, and (k) extracellular vesicle biogenesis score. Note that cells with either high score or expression value cluster on the same portion of the curve (ellipses). Pathifier tool

ELOVL2 is a fatty acid elongase involved in the elongation of 22- to 24-carbon VLC-PUFA [29]. In agreement with *ELOVL2* overexpression in Tum^HIGH^ cells, we observed *ELOVL2* overexpression in cells with tumorigenic properties in an independent transcriptome dataset of human GBM cells in culture [40] (Additional file 3: Figure S7A). In addition, we observed a higher expression of *ELOVL2* in GBM than in normal brain tissues (Additional file 3: Figure S7B), as well as in single GBM cells compared to single normal cells (Additional file 3: Figure S7C). Its expression was also higher in primary GBMs characterized by a wild-type form of *IDH1* compared to diffuse glioma that are characterized by a mutant form of *IDH1* (Additional file 3: Figure S7D). Of note, *ELOVL2* levels were also higher in GBMs bearing an amplified *EGFR* gene than in GBMs with non-amplified *EGFR* (Additional file 3: Figure S7E). Finally, *ELOVL2* high expression was found to be associated with worse patient survival in independent patient cohorts (Fig. 5a and Additional file 3: Figure S7F). Knocking down *ELOVL2* expression in patient-derived cells (PDC) using lentiviral transduction of small hairpin (sh) RNA (Fig. 5b and Additional file 3: Figure S8A) resulted in a sharp decrease in cell proliferation (Fig. 5c and Additional file 3: Fig. S8B). *ELOVL2* role in the control of GBM cell tumorigenicity was evaluated in vivo using orthotopic xenografts of PDC stably expressing luciferase and either shControl or sh*ELOVL2*. Tumor development monitoring with bioluminescent imaging showed delayed tumor formation and reduced tumor burden in mice grafted with sh*ELOVL2*-PDC, compared to mice grafted with shControl-PDC (Fig. 5d and e). Of note, tumors that developed in a delayed manner from xenografts of sh*ELOVL2*-PDC had escaped from *ELOVL2* inhibition, as shown by QPCR detection of human *ELOVL2* mRNA levels at similar levels to those measured in tumors developing from xenografts of shControl-PDC (Fig. 5f). To gain insight into the cell process affected by *ELOVL2*, we selected from the 27 genes of the lipid metabolism, a lipid subgroup of 16 genes detected in at least 25% of the cells (Additional file 9). These genes were used to construct a principal curve onto which each GBM cell was projected (Fig. 5g). In addition, we computed a score with the expression of these 16 genes, following the same procedure as for the tumorigenic score. Of note, highest lipid scores (Fig. 5h), highest *ELOVL2* expression values (Fig. 5i) and highest tumorigenic scores (Fig. 5j) all coincided in cells along the principal curve. This result further strengthens the relationship between the expression of the lipid subgroup, *ELOVL2* expression and the tumorigenic state of the cells. Another member of the ELOVL family, ELOVL4, was involved in extracellular vesicle formation and release [31]. This prompted us to determine whether *ELOVL2* expression is associated with molecular signatures of extracellular vesicles at the single cell level. Scores calculated for each of the four molecular signatures associated with extracellular vesicles were correlated with the tumorigenic scores (Additional file 11) and coincided with high *ELOVL2* expression in cells along the principal curve (Fig. 5k and Additional file 3: Figure S9A-C). Using nanoparticle tracking analysis, we also observed a reduction in the proportion of small-sized extracellular vesicles (50-250 nm) in the culture media of sh*ELOVL2*-PDC compared to shControl-PDC (Additional file 3: Figure S9D-E). This experimental set of results demonstrates that *ELOVL2* is required for the tumorigenic behavior of GBM cells. It also suggests that *ELOVL2* requirement stems from its involvement in the regulation of intercellular communication via extracellular vesicles. In addition, it provides robust experimental support for the relevance of signature-driven reduction of single cell transcriptomes to decipher the metabolic pathways underscoring GBM cell behaviors within the patients’ tumors.

## Discussion

Metabolism is at the heart of cell behavior and its dependence on enzymatic activities makes it a target of choice for therapeutic targeting. GBM cells adapt their functioning state to changes in their microenvironment, whether these changes result from natural tumor growth or therapies. Progress towards identification of the most relevant possible targets for patient therapy requires access to molecular players active in the actual context of the patient tumors while taking into account the heterogeneity of the tumor tissue. Transcriptomes of single cells sorted from patient GBMs offer such an access.

Unsupervised analysis of scRNA-seq from brain and non-brain tumors was found to result in predominant grouping of cancer cells according to the tumor from which they originate, and of normal cells independently from their tumor of origin [11, 14, 23, 35, 39, 57, 64]. This mode of grouping obtained with different normalization and analytical methods was never questioned. We considered in all ways we thought eventual influences of scRNA-seq technical biases as well as of the tumor-specific differences in gene repertoires expected to reflect inter-tumor biological differences whatever their sources. We did not find one accounting for cell grouping-dependence on the tumor of origin. In contrast, unsupervised analyses of down-sampled numbers of genes randomly selected showed that tumor-driven cell grouping disappears below a critical number of genes (100 to 50 in this study). Collectively, our results show that cancer cell clustering per tumor is not due to scRNA-seq technical bias, as expected with respect to the lack of influence of the tumor on normal cell grouping. They also show that cancer cell clustering per tumor is not driven by circumscribed gene subsets reflecting inter-tumor biological differences. They support the notion that the identity of the tumor to which each cancer cell belongs is encoded by information dispersed throughout the cell transcript repertoire.

To unmask cell functioning states regardless of their tumor of origin, we reduced the data based on previously acquired biological knowledge. Signature-driven data reduction was previously used to infer cell lineages from single cell transcriptomes of oligodendrogliomas and diffuse infiltrative pontine gliomas [23, 65]. These studies were performed on centered data. Cell lineages were inferred using sets of top correlated genes to the principal component scores of a PCA of the dataset under scrutiny [65], or on the basis of mouse or human gene sets differentiating normal neural subtypes [23, 65]. Here, we based our analysis on a molecular signature related to a major functioning state, tumorigenicity.

Cells from a given GBM have long been known to be endowed with differing abilities to initiate neoplasms [32, 56, 70]. We postulated that such a choice would enhance the likelihood to group cells based on their functioning state irrespective of their tumor of origin. We postulated also that using a signature defined [4] and experimentally validated in independent prior studies [4, 30, 43, 63] would reduce the risk of obtaining results relevant only for the dataset analyzed. Our choices proved fruitful to identify two contrasting functioning states, with respect to the following findings. Genes previously described as controlling GBM cell aggressiveness were found to be overexpressed in cells with high tumorigenic scores. A highly similar list of differentially expressed genes was obtained when applying the same analytical strategy to an independent dataset of GBM cell scRNA-seq and of another made of tissue bulk transcriptomes of a much larger number of GBMs. Highest scores for the lipid subgroup found to be enriched in Tum^HIGH^ cells and tissues coincided with highest tumorigenic scores. Finally, experimental knockdown of the most interconnected gene of this lipid metabolism subset, *ELOVL2*, impaired GBM cell tumorigenic properties.

Oncogenic mutations have long been known to favor mobilization of metabolic pathways, most notably by allowing cancer cells to adapt their mode of energy production to different microenvironments and sources of nutrients [42, 55]. GBM cells, like other cancer cell types, are considered to favor glycolysis over oxidative phosphorylation for ATP production. This prevalence of glycolysis is considered as a cell adaptation to its need for feeding carbon into biosynthesis of nucleic acids, fatty acids and proteins for cell growth and proliferation. At the single cell level, overexpression of genes coding for enzymes of the glycolysis pathway was not associated with high tumorigenic scores. This suggests that enhanced mobilization of glycolysis enzymes is not proper to GBM cells in a tumorigenic state, at least at the transcriptional level. Overexpression of two genes coding for enzymes of the amino acid pathways, GATM and CKB (Fig. 4b) rather suggests that another source of energy distinguishes tumorigenic from non-tumorigenic GBM cells. GATM is one of the two enzymes ensuring brain-endogenous synthesis of creatine, starting from glycine. The other enzyme, GAMT, was found to be overexpressed in tumorigenic high GBM cells but not retained for final analyses because absent from the list of genes overexpressed in tumorigenic high GBM tissues. CKB is responsible for the phosphorylation of creatine, which serves for ATP regeneration and plays an essential role in brain energy metabolism [59]. Interestingly, GATM is the most interconnected of the genes coding for elements of amino acid metabolism in the modeling of the gene expression network (Fig. 4c). Lipids are also a significant source of energy. Lipid metabolism association to GBM cell aggressiveness has been reported to stem from lipid contribution to cell energetics through fatty acid beta-oxidation and to transduction pathways through the mevalonate metabolism [42]. Accordingly, we found that genes coding for key enzymes of the fatty acid synthesis (e.g. *ACLY*, *FASN*) and beta-oxidation pathways (*CPT1C*), as well as the mevalonate pathways (*MVD*) were overexpressed in Tum^HIGH^ cells. In addition, we observed an overexpression of genes involved in the synthesis of phospholipids, glycerolipids and sphingolipids, essential components of plasma membranes and/or sources of potent autocrine/paracrine signaling molecules.

The biological relevance of the results of our bioinformatics analyses was further validated in in vivo experimental models of human GBMs. Our study unveiled an unexpected causal link between ELOVL2, the endpoint enzymatic component of the lipid subpathway ensuring synthesis of VLC-PUFA, and the tumorigenic status of GBM cells. Notably, we show that *ELOVL2* knockdown in PDC decreases tumor growth in vivo. Little is known on this member of the ELOVL family that catalyzes the elongation of saturated and monounsaturated VLC-FA (ELOVL1, 3, 6 and 7) and of VLC-PUFA (ELOVL2, 4 and 5) by adding two carbon units to the carboxyl end of a fatty acid chain [29]. ELOVL2 is specifically involved in the elongation cascade starting from the dietary PUFA linoleic and linolenic acids, which cannot be synthesized by humans. The products of the enzymatic process of these essential PUFA are thought to modulate diverse biological phenomena ranging from cell survival to inflammatory responses [3, 10]. In mouse models, *Elovl2* knockout has been shown to result in defective PUFA composition in the liver, serum and testis in association with male infertility and a reduced capacity to accumulate fat [54, 74]. PUFA are structural components of membrane phospholipids especially enriched in neural tissues, and provide potent signaling compounds [41]. In the MCF7 cell line that models breast cancers requiring estrogen for growth, *ELOVL2* expression is positively regulated by estrogen [28], and its knockdown is associated with epithelial to mesenchymal transition [38]. In this epithelial cancer high *ELOVL2* expression is associated with higher metastatic relapse-free survival [38]. On the opposite, *ELOVL2* upregulation in prostate cancer has been associated with the oncogenic effect of *SPOP* loss of function mutations [72]. Here, finding of the association between high *ELOVL2* expression in GBMs and worsened patient prognosis (Fig. 5a and Additional file 3: Figure S7F), coupled with the demonstrated requirement of *ELOVL2* for GBM cells tumorigenicity in vivo, demonstrates a causal link between *ELOVL2* and GBM growth. We investigated through bioinformatics analysis what might be the mechanism of action of *ELOVL2* overexpression in tumorigenic high cells. The correlation between *ELOVL2* overexpression and molecular signatures of extracellular vesicles suggests that formation and release of extracellular vesicles is one of the cell processes by which ELOVL2 controls GBM tumor development. This possibility is coherent with the reported involvement of another member of the family, *ELOVL4*, in the formation of synaptic vesicles in the brain and retina [31]. Extracellular vesicles have been involved in intercellular communications within GBM, by carrying metabolites, nucleotides and proteins able to affect the behavior of cancerous as well as non-cancerous cells composing the tumor [6, 22]. Our experimental results are coherent with our modeling results that place ELOVL2 at the core of the metabolic pathways essential for sustaining GBM cell tumorigenicity. ELOVL2 importance for GBM is strengthened by a study published during writing of this article that describes *ELOVL2* as a super-enhancer associated gene controlling glioblastoma stem cell properties [27].

## Conclusions

The present findings underscore the power of single cell transcriptome analyses for unveiling the complexity of the participation of metabolism in relation to the heterogeneity of cell functioning states encountered in GBMs. It is worth emphasizing that the discovery of a molecular deregulation that proved to be a predictor of patient survival in independent cohorts of several hundred tumors stems from the study of cells derived from only four tumors. Our results show the high relevance of integrating the cell functioning status, even when focusing on only two contrasting states, for the discovery of metabolic modules controlling GBM aggressiveness. Further development of signature-driven data reduction based on established experimental evidence will lead to further refine the identification of functioning states and of the diversity of the molecular networks required for their maintenance.

## Supplementary information

**Additional file 1:** Supplementary Material and Methods.

**Additional file 2:** List of all resources and materials, R packages, corresponding websites and references.

**Additional file 3: Figure S1.** Removing cells with low-complexity transcriptomes and unsupervised grouping analysis of GBM and normal cells simultaneously. **Figure S2.** Maintenance of tumor-driven cell grouping regardless of the mode of data normalization or filtering. **Figure S3.** Numbers of transcripts and genes per Tum^HIGH^ versus Tum^LOW^ cell. **Figure S4.** Signature-based analytical workflow. **Figure S5.** Main metabolic pathways containing the metabolic enzyme genes overexpressed in Tum^HIGH^ GBM cells and tissues. **Figure S6.** Similar metabolic modules distinguish Tum^HIGH^ and Tum^LOW^ GBM cells independently from *EGFR* amplification. **Figure S7.** Increased *ELOVL2* expression is associated with increased tumor burden. **Figure S8.** Knocking down *ELOVL2* expression using a second sh *ELOVL2* construct. **Figure S9.** GBM cell tumorigenic state and extracellular vesicle production and release.

**Additional file 4:** R scripts used for unsupervised grouping and associated analyses.

**Additional file 5:** GO analysis of genes describing the seven clusters identified upon grouping analysis using standardized data.

**Additional file 6:** List of housekeeping genes used for data normalization.

**Additional file 7:** Genes differentially expressed between low and high tumorigenic GBM cells and/or tissues.

**Additional file 8:** R scripts used for signature-based analytical workflow.

**Additional file 9:** Correlation of the metabolism genes overexpressed in Tum^HIGH^ cells and tissues with the tumorigenic score.

**Additional file 10:** Overexpressed metabolism genes common to Neftel and Darmanis Tum^HIGH^ cells and to Tum^HIGH^ GBM tissues.

**Additional file 11:** Correlation across all cells between the tumorigenic score, *ELOVL2* expression, and the extracellular vesicle-related scores.

## Abbreviations

BH: Benjamini-Hochberg
CNV: Copy Number Variations
CPM: Counts Per Million
GBM: Glioblastoma
HCPC: Hierarchical Clustering on Principal Components
HKG: HouseKeeping Genes
MIIC: Multivariate Information-based Inductive Causation
NMI: Normalized Mutual Information
PCA: Principal Component Analysis
PDC: Patient-Derived Cells
scRNA-seq: single cell RNA-sequencing
shRNA: small hairpin RNA
tSNE: t-distributed Stochastic Neighbor Embedding
Tum: Tumorigenic
VLC-PUFA: Very Long Chain PolyUnsaturated Fatty Acids
