# Supplementary material for: Capture at the single cell level of metabolic modules distinguishing aggressive and indolent glioblastoma cells
Source: Acta Neuropathol Commun. 2019 Oct 16;7:155. doi: 10.1186/s40478-019-0819-y (PMC6796454; doi:10.1186/s40478-019-0819-y)
Supplement: Supplementary file 1 — Additional file 1: Supplementary Material and Methods. [file 40478_2019_819_MOESM1_ESM.pdf]

Additional file 1 (related to Material and Methods).

Public data acquisition

The single cell transcriptomes of 1091 GBM cells and 2498 normal cells originating from four patients were downloaded from http://gbmseq.org/ [14]. This dataset comprises only good-quality cells, as determined by its authors on the basis of the expression of 35 housekeeping genes (HKG), and distinguishes cancer from normal cells on the basis of CNV profiling. Normal cells are further distinguished according to their neural or immune lineage subtype [14]. Normalized counts in $\log_2$(TPM+0.5) from TCGA RNA-seq data of 155 untreated GBM patients with available survival data were obtained from the Gliovis portal (http://gliovis.bioinfo.cnio.es/). We also used a dataset of single cell transcriptomes published during the reviewing process of this article (https://portals.broadinstitute.org/single_cell), using expression values in log2(TPM+1) [51]. It comprises data from good-quality cells i.e. 4916 GBM cells derived from twenty adult patients.

Standardization or normalization of single cell transcriptome data

For analyses of single cell transcriptomes, we used log2-transformed Counts Per Million ($\log_2$(CPM+1)), unless otherwise specified. CPM corresponds to the counts of gene mapped reads normalized by the total number of mapped reads per cell library divided by one million, thus allowing comparison of read abundance across libraries of different sizes. To avoid potential analytical bias due to scarcely detected genes, we filtered out genes detected in less than 3 cells prior to grouping analysis, keeping 18577 genes for GBM cells and 19699 genes for normal cells. When grouping analysis included both GBM and normal cells, we kept the 20237 genes detected in at least 3 GBM cells or 3 normal cells.

In a subset of analyses, tumor-per-tumor data standardization was achieved by centering and reducing the data on a gene-by-gene basis as described: $x_{Ti}' = (x_{Ti} - \text{mean}(x_T)) / \text{sd}(x_T))$, where x_{Ti} corresponds to a gene transcript level in a given cell from a given tumor T, and mean(x_T) and sd(x_T) to the arithmetic mean and standard deviation across GBM cells of tumor T, respectively [8, 45]. Note that grouping analysis using data standardized by tumor resulted in 10 clusters, including 3 with only 1 cell each. These 3 clusters were ignored in further analyses.

In another subset of analyses, data were normalized on the basis of the expression of a set of housekeeping genes (HKG). The 35 genes considered by Darmanis *et al* as HKG were not used because of their variable detection in 2.13 to 99.9% GBM cells and variable expressions (coefficients of variation (CV) ranging from 0.10 to 7.93). Here, we defined HKG according to the following criteria: genes detected in most cells, with relatively constant expression across all cells and whose expressions auto-correlate. Thus, calculation of the Pearson correlation (corr) between each pair of genes was followed by calculation of the distance matrix between each pair of genes based on their correlation matrix (1-corr). Groups of genes with correlated expressions were identified using a hierarchical clustering (Ward's method using ward.D2 algorithm, cutting dendrogram into clusters at h=1.2). Next, we selected the gene group with the smallest average CV of gene expression. From this group, we retained only genes detected in at least 90% cells, defining thus a set of 17 HKG expressed with a CV of their expression ranging from 0.19 to 0.42. To normalize data according to HKG, we first defined small groups of 3 to 65 cells with similar expression profiles, using the Pearson correlation between each pair of cells and the corresponding distance matrix (1-corr), followed by hierarchical clustering (Ward's method using ward.D2 algorithm, with h varying between 0.8 and 1.1 to obtain clusters with less than 65 cells). The arithmetic mean expression of each HKG was calculated for each of these small cell groups considered as metacells. The normalization factor corresponded to the geometric mean of all HKG per metacell (psych R package for geometric mean calculation). The data were normalized by dividing each gene expression value per cell by the normalization factor of its corresponding metacell.

We also used the scran method specifically designed for normalization of scRNA-seq data [66]. We obtained log-normalized expression values by combining the function "normalize" of the scater R package where the count is divided by a size factor computed by the "computeSumFactor" function of scran and then log transformed. An additional step to sum factor computing is cell clustering in order to apply the deconvolution method to each cluster (metacell) instead of all cells. To do so, we used the scran function "quickCluster" using each method (hclust and igraph) where clusters must have a minimal size of 100 cells. We presented HCPC results obtained with data normalized using scran after calculating the size factor without a clustering step (designated as C for "Across all cells" on Fig 1d and

Additional file 3: Fig. S2B), or per metacell defined upon hierarchical clustering (designated as MC_H on Fig 1d and Additional file 3: S2C) or per metacell defined upon SNN (Shared Nearest Neighbouring, igraph method) clustering (designated as MC_S on Fig 1d and Additional file 3: S2D). The tumorigenic signature-based analysis was done with data normalized using scran after calculating the size factor across all cells (Additional file 7, sheet 5).

Grouping analyses

Grouping analyses were performed using the Hierarchical Clustering on Principal Components (HCPC) approach (FactoMineR package). This approach combines three standard methods for multivariate data analyses performed stepwise: Principal Component Analysis (PCA), hierarchical clustering, and partitioning clustering by the k-means method (http://factominer.free.fr/more/HCPC_husson_josse.pdf). PCA was performed to reduce the dimensions to the first 10 principal components (PCs). Euclidean method was used to construct a cell-to-cell distance matrix. Hierarchical clustering was then performed on this distance matrix using the Ward's criterion (ward.D2 algorithm). The resulting partitioning of the cells was improved by a K-means clustering with 10 iterations. The cell grouping was visualized using PCA (FactoMineR package), tSNE implemented using the tsne R package (perplexity = 50) or chord plots (circlize R package). In addition, HCPC allows identifying the variables that drive cell grouping for each cell cluster, i.e. genes whose mean expression in one cluster differs from its mean across all cells (pval < 0.05, t-test).

A Normalized Mutual Information (NMI) score was calculated to determine the contribution of cells issued from distinct tumors to each cluster (ClusterR package). This metric is used as an external clustering validation that compares clustering results to a known truth [46]. A NMI value of 1 implies that clusters gather objects (cells) corresponding to a single label (here, the tumor label), whereas a value of 0 denotes that all labels are split across all clusters. Graphs were generated with Prism 7.0 software (GraphPad).

Differential gene expression analysis

Differential gene expression analyses between differing cell or tissue groups were performed using the Mann-Whitney (Wilcoxon Rank Sum) test [62]. The p-values were adjusted for multiple testing using Benjamini-Hochberg (BH) approach. The level of significance was set at BH-adjusted p-value < 0.01. Fold change (FC) for gene i was calculated as follows: $FC_i=x_i-y_i$, where x_i and y_i are the log2 expression levels of gene i in conditions x and y, respectively. Only genes detected in at least 3% of GBM cells or tissues were considered for this analysis. Genes coding for metabolism enzymes were identified using the list from KEGG [37].

Gene ontology analysis

Functional enrichment analysis was carried out using the online database DAVID v6.8 (https://david.ncifcrf.gov/). The human genome was used as background (Homo Sapiens from DAVID). We considered p-value < 0.05 (Fisher's Exact test) as the cut-off criterion for significance. Graphs were generated with Prism 7.0 software (GraphPad).

Functional gene network reconstruction

Prior to the network reconstruction, expression data were binarized into an ON-OFF system. For each cell, detected genes were considered as ON and assigned a value of 1 regardless of their relative expression levels. Undetected genes were considered as OFF and retained their original value of 0.

The information-theoretic method, MIIC (multivariate information-based inductive causation), was used for gene network reconstruction and performed on the dedicated website (https://miic.curie.fr/) [61, 69]. This method is based on the analysis of multivariate information, which extends the concept of mutual information beyond two variables and may imply cause-effect relationships between the underlying variables. On the resulting graph, the edge between 2 variables X and Y reflects their connection with an edge-specific confidence ratio <0.01. The lower the ratio, the higher the confidence.

Cell cultures

Patient-derived cells (PDC) 6240**, R633 and 5706** obtained from neurosurgical biopsy samples of distinct primary GBM were cultured in defined medium containing bFGF and EGF as described [19, 60]. The cells were transduced with lentiviral vectors encoding a control or an ELOVL2 shRNA construct (pLKO.1-HPGK-puro-U6-non mammalian shRNA control, and pLKO.1-puro-CMV-tGFP-U6-shELOVL2-61 and pLKO.1-puro-CMVtGFP-U6-shELOVL2-64, Sigma, France). Non-transduced cells were eliminated following puromycin treatment (2 µg/mL). Lentivirus was produced by the Plateforme vecteurs viraux et transfert de gènes (Necker Federative structure of research, University Paris Descartes, France).

Viable cell counting

Trypan blue exclusion test was used to determine the number of viable cells (Trypan blue solution, ThermoFisher, 0.4% v/v, 3 min incubation at room temperature). Blue and white cells (dead and alive, respectively) were counted with the Countess automated cell counter (Thermo Fisher, France). Cell proliferation was determined after completing the selection step of transduced cells with a 10-day treatment with puromycin. Cells transduced with either shControl or sh*ELOVL2* were then seeded in fresh defined medium and viable cell numbers evaluated 5-7 days later.

Extracellular vesicle analysis

Extracellular vesicles were obtained from the culture media of 6240** PDC transduced with shControl or sh*ELOVL2* (n=3, each), seeded at a density of 600000 cells/mL in a 12-well plate and cultured without puromycin for 4 days. Either cell condition had a similar proportion of viable cells at the time of extracellular vesicle harvesting (% of viability shControl cells 94.7±1.2, sh*ELOVL2* cells 85.7±6.0, mean±SD, n=3). The supernatants were collected after centrifugation (233g, 5 min, 21°C). They were then submitted to two successive centrifugation steps (400g, 10 min, 4°C and 2000g, 10 min, 4°C) to eliminate cell debris, prior to be stored at -80°C. Nanoparticle tracking analysis was performed on the NS300 system (Nanosight, Malvern Panalytical) using extracellular vesicles from culture cell media as described [17, 26]. This technique evaluates in solution individually and simultaneously nanoparticles ranging from 10nm to 1000nm by direct observation and measurement of diffusion events, thus providing a measure of nanoparticle size distribution and concentration. The measures were performed on 5 videos of 1min for each sample, and the data normalized to the number of cells.

Gene expression analysis

Total RNA was prepared using the Nucleospin RNA kit (Macherey-Nagel) according to the manufacturer's instruction. Contaminating DNA was removed using an rDNAse solution supplied with the Nucleospin RNA kit during RNA isolation. cDNA was prepared using the QuantiTect Reverse Transcription Kit (Qiagen) according to manufacturer's instructions. QPCR assays were performed using the LightCycler480 (Roche, France) and the SYBR Green PCR Core Reagents kit (Bimake.com). The thermal cycling conditions comprised an initial denaturation step at 94 °C for 5 min, and 40 cycles at 94 °C for 30 s, 60 °C for 30 sec and 72 °C for 30 sec. Transcripts of the TBP gene encoding the TATA box-binding protein (a component of the DNA- binding protein complex TFIID) were quantified as an endogenous RNA control. Quantitative values were obtained from the cycle number (Cq value), according to the manufacturer's manuals. Sequences of primers used for QPCR are: ELOVL2 - forward primer: 5'-TCCACTTGGGAAGGAGGCTACA; ELOVL2 - reverse primer: 5'-CTCCAAATCAGTAGAGTTCCTGG; TBP - forward primer: 5'-TGCACAGGAGCCAAGAGTGAA; TBP - reverse primer: 5'-CACATCACAGCTCCCCACCA.

Intracranial xenografts

The animal maintenance, handling, surveillance and experimentation were performed in accordance with and approval from the Comité d'éthique en expérimentation animale Charles Darwin N° 5 (Protocol #5379). 6240** and 5706** PDC transduced with lentiviruses encoding either a shControl or a sh*ELOVL2*, were used. 20000 cells (6240**) or 100000 cells (5706**) were injected stereotaxically into the striatum of anesthetized 8-week-old Nude mice (Envigo Laboratories, France), using the following coordinates: 0 mm posterior and 2.5 mm lateral to the bregma, and 3 mm deep with respect to the

surface of the skull. Bioluminescence imaging was performed on a Photon Imager Biospace (Biospace Lab, France), after intra-peritoneal injection of 150 µL luciferin (20 mM, Thermo Fisher, France). Tumor formation was monitored by bioluminescence until all mice of the control group showed a signal. Bioluminescent signals were visualized with M3 Vision software (Biospacelab).
