# Supplementary material for: Capture at the single cell level of metabolic modules distinguishing aggressive and indolent glioblastoma cells
Source: Acta Neuropathol Commun. 2019 Oct 16;7:155. doi: 10.1186/s40478-019-0819-y (PMC6796454; doi:10.1186/s40478-019-0819-y)
Supplement: Supplementary file 3 — Additional file 3: Figure S1. Removing cells with low-complexity transcriptomes and unsupervised grouping analysis of GBM and normal cells simultaneously. Figure S2. Maintenance of tumor-driven cell grouping regardless of the mode of data normalization or filtering. Figure S3. Numbers of transcripts and genes per TumHIGH versus TumLOW cell. Figure S4. Signature-based analytical workflow. Figure S5. Main metabolic pathways containing the metabolic enzyme genes overexpressed in TumHIGH GBM cells and tissues. Figure S6. Similar metabolic modules distinguish TumHIGH and TumLOW GBM cells independently from EGFR amplification. Figure S7. Increased ELOVL2 expression is associated with increased tumor burden. Figure S8. Knocking down ELOVL2 expression using a second sh ELOVL2 construct. Figure S9. GBM cell tumorigenic state and extracellular vesicle production and release. [file 40478_2019_819_MOESM3_ESM.pdf]

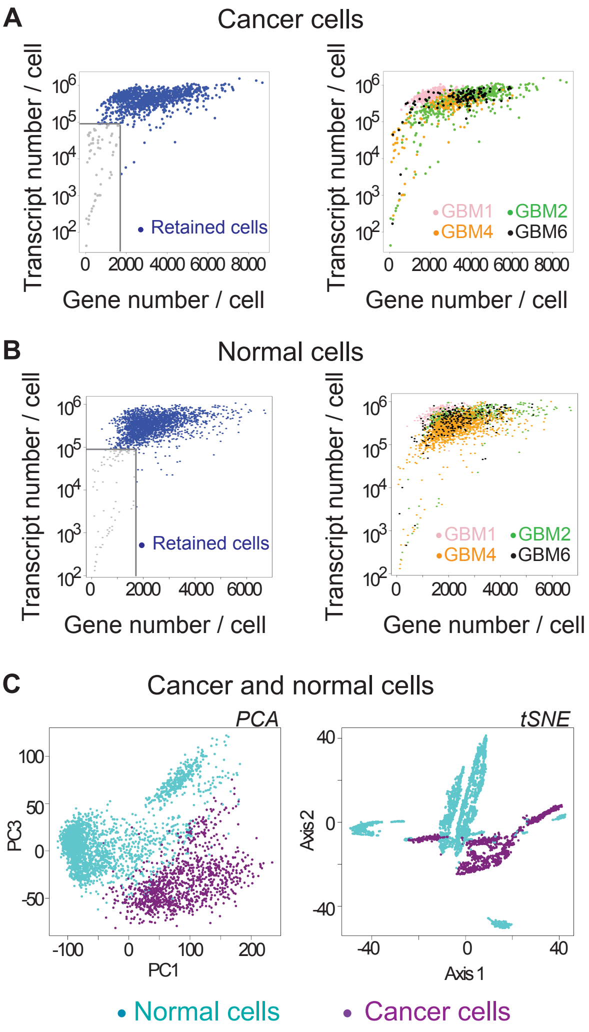

Fig. S1 (related to Fig. 1). **Removing cells with low-complexity transcriptomes and unsupervised grouping analysis of GBM and normal cells simultaneously.** **A-B.** Filtering out cells with low-complexity transcriptomes. Cells with more than 90000 transcripts and more than 1700 genes were selected for further analyses. 1033 GBM (A) and 2417 normal cells (B) were retained. Left panels: selected cells colored in blue, rejected cells colored in gray. Right panels: cells colored by tumor. **C.** Unsupervised grouping analysis of the mixed set of GBM and normal cells distinguishes cancer cells from normal cells. Each dot represents a cell. Normal cells colored in light blue, cancer cells colored in violet. Left panel: PCA visualization. Right panel: tSNE visualization.

Saurty-Seerunghen MS et al. Capture at the single cell level of metabolic modules distinguishing aggressive and indolent glioblastoma cells.

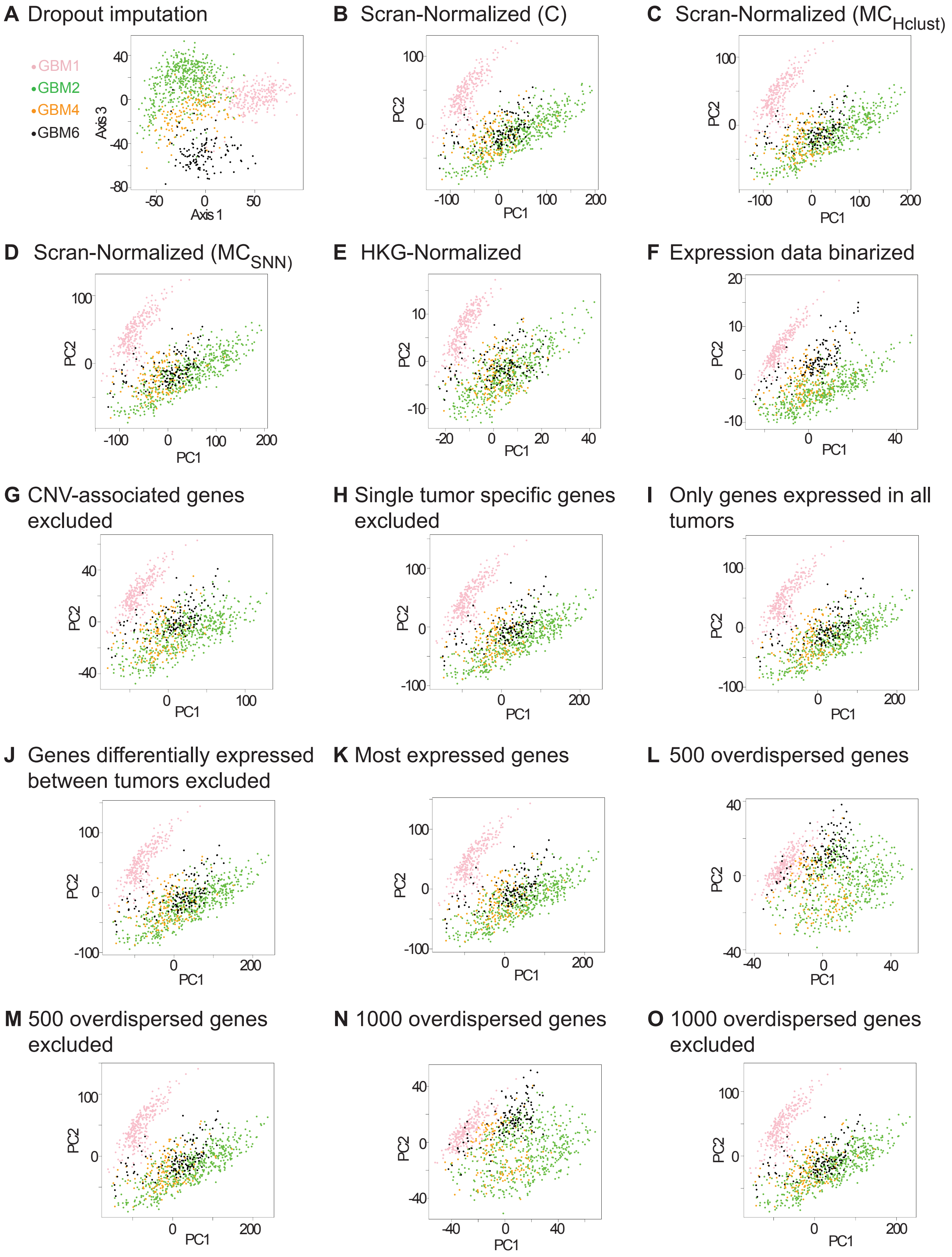

Fig. S2 (related to Fig. 1). **Maintenance of tumor-driven cell grouping regardless of the mode of data normalization or filtering.** A: Multidimensional scaling (MDS) visualization. B-O: PCA visualization. Each dot represents a cell. **A-E.** Technical biases related to scRNA-seq do not contribute to tumor-driven cell grouping. A. Maintenance of tumor-driven cell grouping after imputation of dropouts using the algorithm CIDR (Clustering through Imputation and Dimensionality Reduction). CIDR imputes dropouts by inferring their values from gene expression across all cells [44]. B-D. Normalizing data by a size factor calculated across all cells (B) or per metacell (MC) defined using hierarchical (C) or SNN-based (D) clustering does not change tumor-driven cell grouping.
E. Normalizing data by the expression of housekeeping genes (HKG) prior to analysis fails to alleviate tumor-driven cell grouping.
F-O. Tumor-specific biological differences are not reducible to circumscribed sets of genes. F. Binarization of expression data to overcome possible inter-experimental variations in the efficacy of scRNA-seq, and therefore in the detected RNA levels, does not change tumor-driven cell grouping. G. Filtering out genes located on chromosomes with identified variations in copy number (CNV) does not modify tumor-driven cell grouping. H. Tumor-driven cell grouping is maintained after removal of genes detected in a single tumor. I. Grouping analysis performed using only genes detected in all tumors also results in tumor-driven cell grouping.
J. Filtering out genes differentially expressed between tumors does not alleviate tumor-driven cell grouping. K. Cell grouping analysis using the most expressed genes across cells fails to overcome tumor-driven cell grouping. L. Cell grouping analysis using the top 500 genes with the most overdispersed expression across cells fails to overcome tumor-driven cell grouping. M. Filtering out the top 500 genes with the most overdispersed expression across cells does not change tumor-driven cell grouping. N. Cell grouping analysis using the top 1000 genes with the most overdispersed expression across cells fails to overcome tumor-driven cell grouping. O. Filtering out the top 1000 genes with the most overdispersed expression across cells does not change tumor-driven cell grouping.

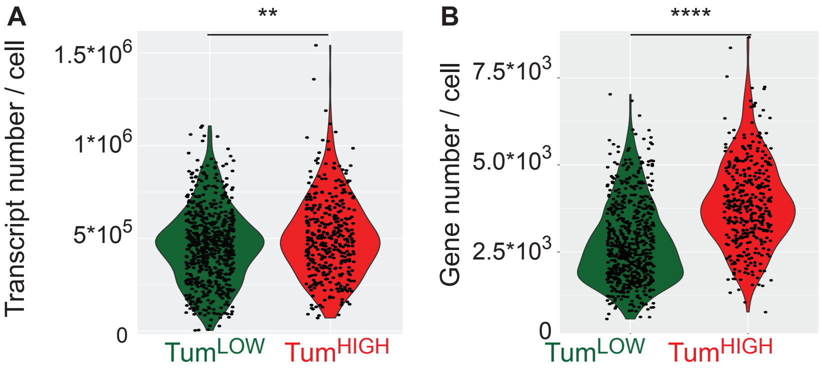

Fig. S3 (related to Fig. 3). **Numbers of transcripts (A) and genes (B) per Tum^{HIGH} versus Tum^{LOW} cell.** Mann-Whitney test. p = 1.92*10-3 for transcript number and 2*10-41 for gene number.

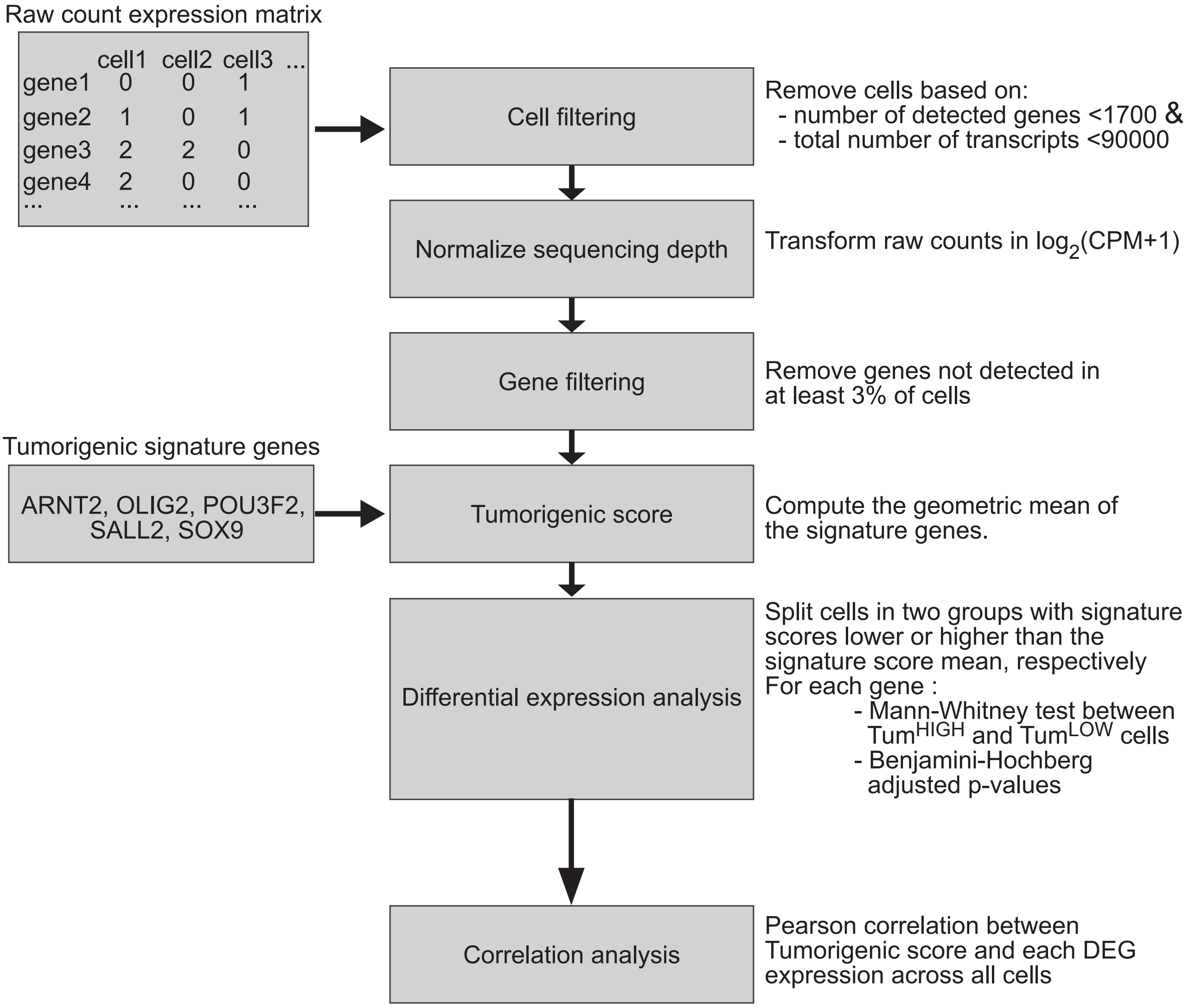

Fig. S4. Signature-based analytical workflow.

The analytical method developed has been implemented in R and is provided in Additional file 8.

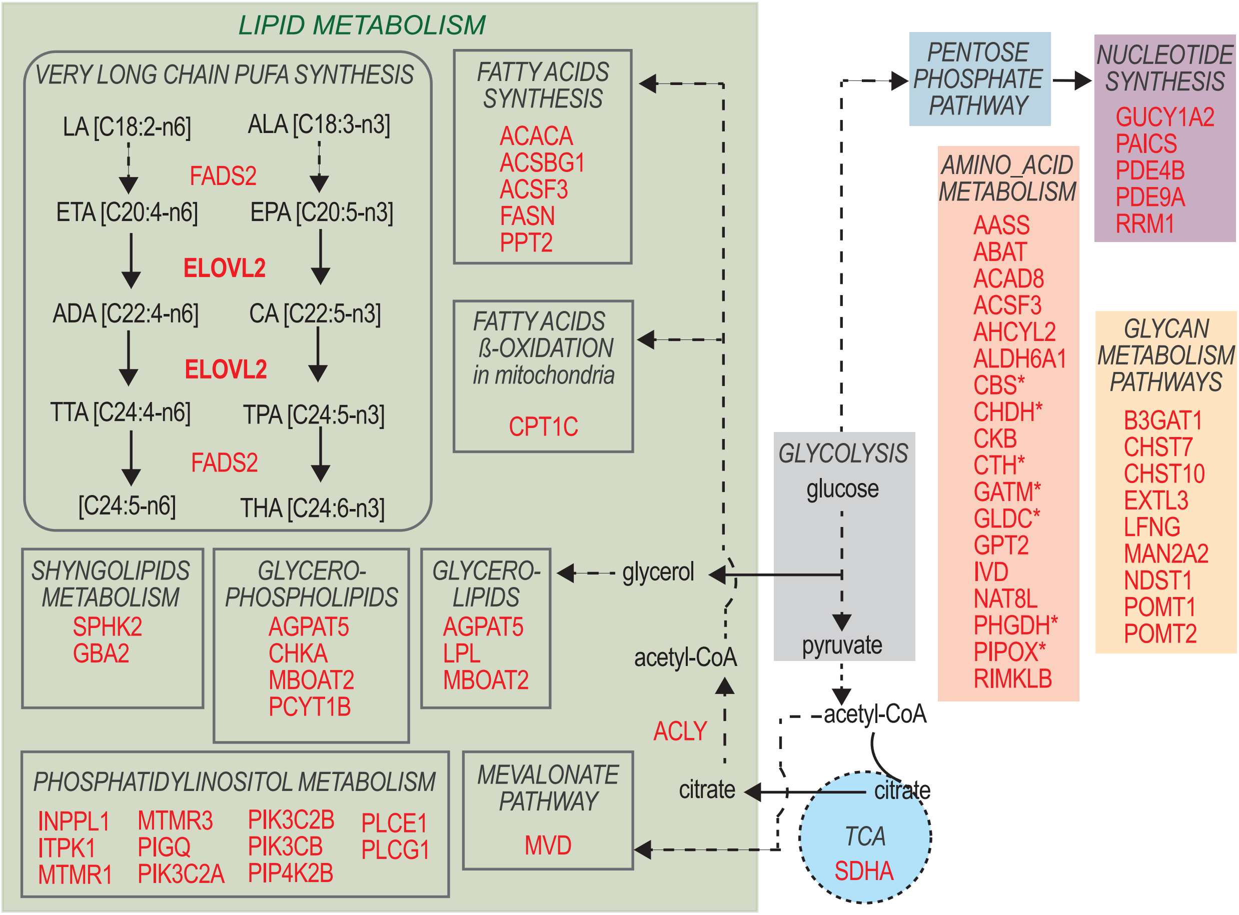

Fig. S5 (related to Fig. 4)
Main metabolic pathways containing the metabolic enzyme genes overexpressed in Tum^{HIGH} GBM cells and tissues. The scheme contains 60 of the 66 metabolic enzyme genes overexpressed in Tum^{HIGH} GBM cells and tissues. Asterisks mark genes coding for components of the glycine, serine and threonine metabolism.

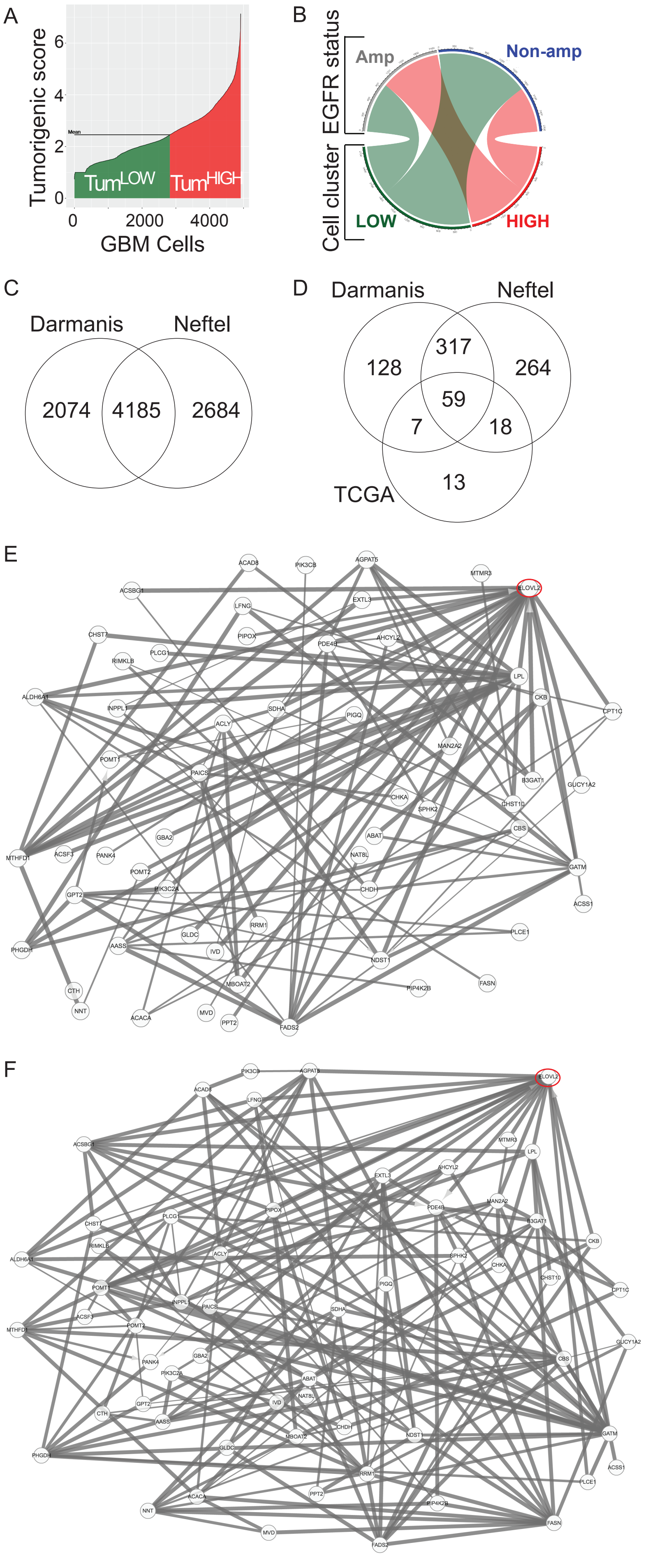

Figure S6

Fig S6 (related to Fig. 3 and 4).
Similar metabolic modules distinguish Tum^{HIGH} and Tum^{LOW} GBM cells independently from EGFR amplification.
A. Tumorigenic score distribution across the cells using the GBM cell scRNA-seq from Neftel and colleagues [Neftel, 2019]. Cells are split into groups with high (Tum^{HIGH}) or low (Tum^{LOW}) tumorigenic potential according to mean of the tumorigenic score (dotted line). **B.** Cells derived from EGFR^{Amp} and EGFR^{NonAmp} GBMs contribute both to the Tum^{HIGH} and Tum^{LOW} GBM cell groups (chord plot). **C.** High overlap between genes overexpressed in Tum^{HIGH} GBM cells from the Darmanis and Neftel datasets. 60.9% (4185) of genes overexpressed in Tum^{HIGH} GBM cells from Neftel dataset are also overexpressed in Tum^{HIGH} GBM cells from Darmanis dataset. **D.** 59 genes coding for metabolism genes identified among genes overexpressed in both GBM Tum^{HIGH} cells and tissues. Comparing genes overexpressed in Tum^{HIGH} GBM cells from the GBM scRNA-seq datasets (Darmanis and Neftel) and TCGA GBM tissues identifies 59 genes coding for proteins involved in metabolism and whose expression correlates to the tumorigenic score. **E-F.** Modeling interconnections between the 59 metabolism genes using either the Darmanis (E) or Neftel (F) dataset highlights *ELOVL2* at the most densely connected nodes of the network (red circle). Gene network built on the basis of the gene expression values across all GBM cells using MIIC tool. Line thickness represents the strength of the edge. Arrowheads linking variables in a v-structure of the type $x \rightarrow y \leftarrow z$ denotes the absence of a graphical structure of the type: $x \rightarrow y \rightarrow z$, $x \leftarrow y \leftarrow z$ and $x \leftarrow y \rightarrow z$ (variable x cannot be reached passing through y, nor y passing through x, nor y is a common parent of the two other variables). These 3 models can be excluded since in a v-structure y does not mediate mutual information between x and z.

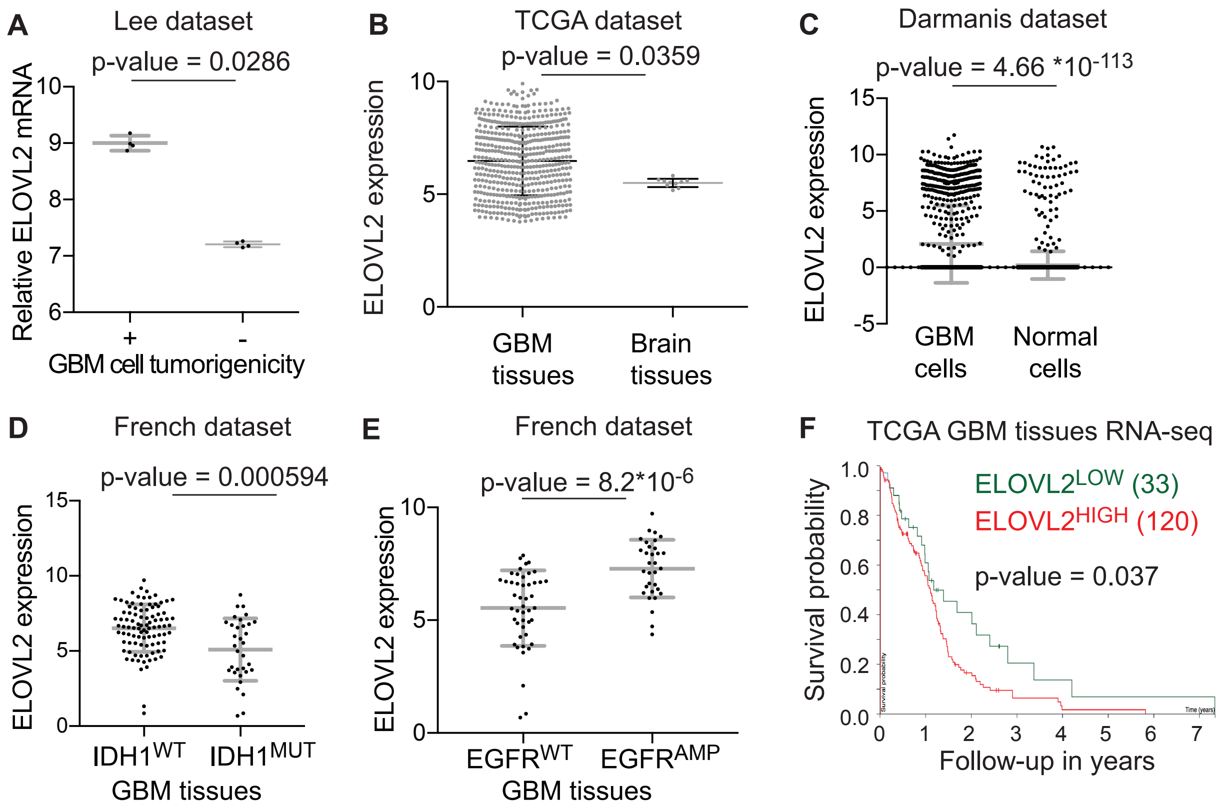

Fig. S7 (related to Fig. 5)
Increased ELOVL2 expression is associated with increased tumor burden.
A. Down-regulated *ELOVL2* expression in patient-derived GBM cells deprived of tumorigenic properties, compared to their tumorigenic counterparts. Mean±SD, Mann-Whitney test. Lee microarray dataset GEO ID: GSE4536.
B. Higher *ELOVL2* expression in GBM tissues compared to normal brain tissues. Mean±SD, Mann-Whitney test. TCGA tissue transcriptome dataset (microarrays) of 528 primary GBM and 10 normal brain tissues.
C. Significantly higher *ELOVL2* expression in GBM cells compared to normal cells. Mean±SD, Mann-Whitney test. scRNA-seq dataset from Darmanis and colleagues [14].
D-E. *ELOVL2* expression is higher in GBM bearing a wild-type form of *IDH1* (D) and with *EGFR* gene amplification (EGFR^{AMP}) (E) than in *IDH1*-mutant and non-amplified *EGFR*, respectively. French tissue transcriptome dataset (microarrays). IDH1^{WT}, n=95. IDH1^{MUT} n=33. EGFR^{WT}, n=46. EGFR^{AMP}, n=32. Mean±SD, Mann-Whitney test.
F. High *ELOVL2* expression is associated with a poorer survival for patients. TCGA GBM tissue transcriptomes (RNA-seq) of 153 primary GBM. Log-rank test.

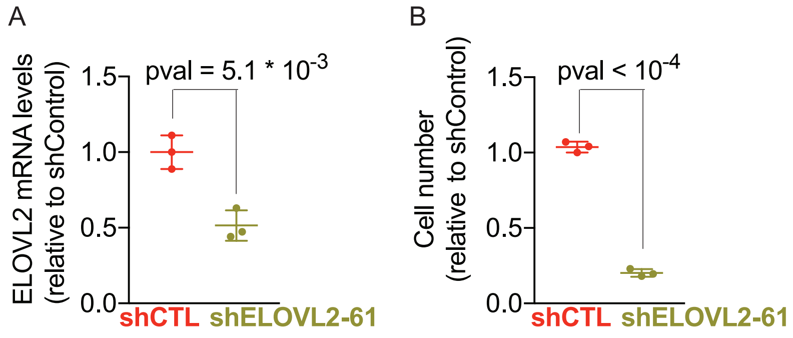

Fig. S8 (related to Fig. 5).
Knocking down *ELOVL2* expression using a second sh*ELOVL2* construct (shELOVL2-61, Sigma, France).
A. Decreased *ELOVL2* mRNA levels in sh*ELOVL2*-61-6240** patient-derived cells (PDC) compared to shControl cells. QPCR assay. Unpaired t-test with Welch's correction, mean ± SD, n = 3 independent biological samples. **B.** *ELOVL2* down-regulation decreases cell proliferation. 6240** cells. Unpaired t-test with Welch's correction, mean ± SD, n = 3 independent biological samples.

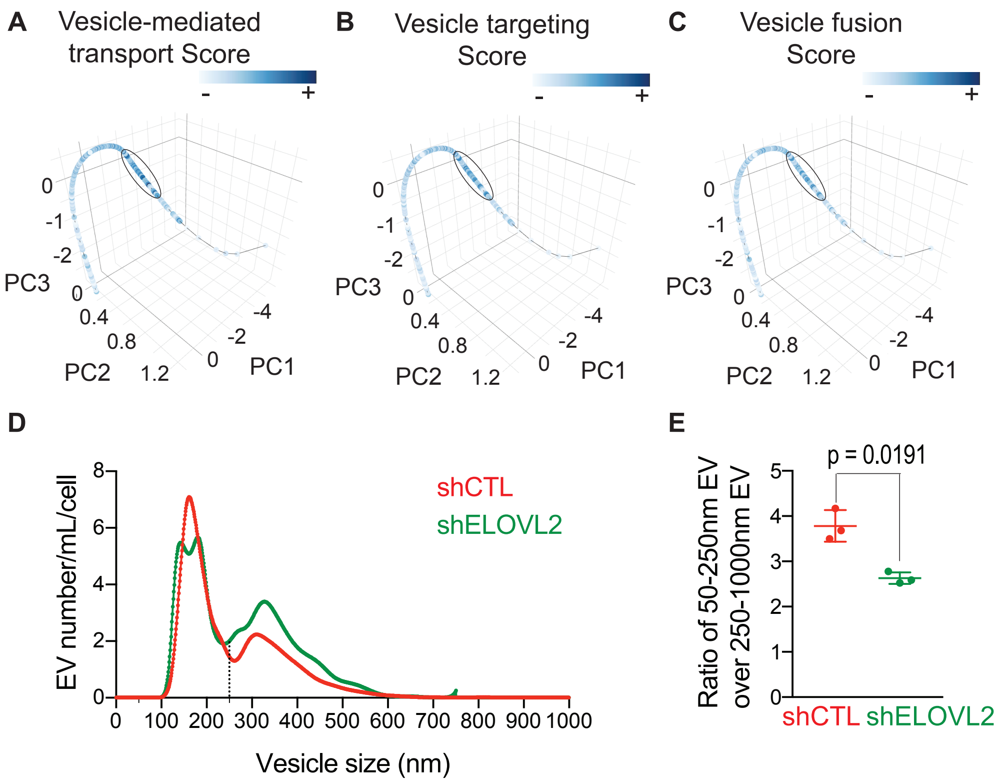

Fig. S9 (related to Fig. 5)**. GBM cell tumorigenic state and extracellular vesicle production and release.**

A-C. Principal curve resulting from PCA of the expression of the subgroup of genes encoding lipid metabolism enzymes overexpressed in Tum^{HIGH} cells and tissues. Cells colored according to their score calculated with the components of molecular signatures associated with extracellular vesicle transport (A)**,** targeting (B), and fusion (C). Note that cells with either high score cluster on the same portion of the curve (ellipses). **D.** Example of the size distributions of extracellular vesicles (EV) collected from the culture media of PDC expressing shControl or sh*ELOVL2*. Dotted vertical line marks the 250 nm threshold used to split vesicles between small and large-sized objects. **E.** Reduction in the proportion of small-sized extracellular vesicles (50-250nm) isolated from sh*ELOVL2*-PDC compared to shControl-PDC. 6240** cells. Unpaired t-test with Welch's correction, mean ± SD, n = 3 independent biological samples.
